# Letter to editor: Multichannel deep learning prediction of major pathological response after neoadjuvant immunochemotherapy in lung cancer: a multicenter diagnostic study

**DOI:** 10.1097/JS9.0000000000003188

**Published:** 2025-08-26

**Authors:** Daiwei Liu, Zhanlin Li, Qiang Ji

**Affiliations:** a Traditional Chinese Medicine Department, The First Affiliated Hospital of Hebei North University, Zhangjiakou, China; b Thoracic and Cardiovascular surgery, The First Affiliated Hospital of Hebei North University

*Dear Editor,*

We read with great interest the recently published study in your journal entitled “Multichannel deep learning prediction of major pathological response after neoadjuvant immunochemotherapy in lung cancer: a multicenter diagnostic study”^[1]^. The authors proposed an innovative approach integrating multi-scale CT features into a deep learning framework, reporting impressive diagnostic performance. While the work presents a novel concept, we would like to raise several methodological and clinical concerns after a careful review. This article was written in accordance with the TITAN guidelines^[2]^.

First, the sample size appears insufficient given the complexity of the model. The authors employed a hybrid architecture combining multi-branch GoogLeNet and Transformer modules, trained on only 172 patients, while extracting a high-dimensional feature set, including 1834 radiomics features. This results in a classic high-dimensional, low-sample-size scenario, which is highly susceptible to overfitting.

Second, the tumor segmentation approach may introduce subjective bias. All regions of interest (ROIs) were manually delineated by two experienced radiologists, yet no assessment of inter-rater reliability or reproducibility was reported. This is particularly concerning given the model’s dependence on ROI input. Additionally, although the proposed Transformer_GoogLeNet model achieved the highest performance (AUC = 0.924), it was not compared against widely adopted architectures such as ResNet, EfficientNet^[3]^, or Vision Transformer^[4]^. This lack of benchmarking raises concerns regarding model selection bias and potential overestimation of performance. Moreover, the study did not include comparisons across single-modality, multi-modality (e.g., PET/CT), or clinical–radiological fusion models, which limits the generalizability of the conclusions.

Third, although clinical variables such as PD-L1 expression, smoking history, and histological subtype were collected, they were not incorporated into the modeling process. Given the multifactorial nature of MPR, relying solely on imaging features may oversimplify the biological and clinical heterogeneity that underlies immunotherapy response.

Fourth, while the dataset included 332 patients from four institutions, both the training and validation cohorts were derived exclusively from a single center (Center 1). The external test set included relatively small sample sizes from the remaining centers (*n* =20 and *n* =19 from Centers 3 and 4, respectively), which may not adequately reflect real-world heterogeneity. Such sample imbalance could hinder the generalizability of the model across different clinical settings. Additionally, variability in CT scanner protocols and image quality across centers warrants further elaboration to ensure consistency and reproducibility.

In summary, this study addresses a timely and important topic at the intersection of AI imaging and immunotherapy. However, improvements in study design, model transparency, and clinical integration are necessary to support its practical utility. We recommend the integration of clinical biomarkers into a multimodal predictive framework, prospective multicenter validation, and the development of decision-impact models in real-world scenarios. We commend the authors for their efforts and encourage further studies to refine and validate the proposed approach for broader clinical adoption.

## Data Availability

Not applicable.
